# Latent tuberculosis infection among undergraduate nursing students: a study of social representations

**DOI:** 10.1590/0034-7167-2025-0282

**Published:** 2026-07-24

**Authors:** Erlon Gabriel Rego de Andrade, Ivaneide Leal Ataide Rodrigues, Laura Maria Vidal Nogueira, Alexandre Aguiar Pereira, Eliza Paixão da Silva, Adriely Alciany Miranda dos Santos

**Affiliations:** I Universidade do Estado do Pará. Belém, Pará, Brazil

**Keywords:** Latent Tuberculosis, Students, Nursing, Social Representation, Psychology, Social, Public Health., Tuberculosis Latente, Estudiantes de Enfermería, Representación Social, Psicología Social, Salud Pública.

## Abstract

**Objectives:**

to analyze nursing students’ social representations regarding latent tuberculosis infection.

**Methods:**

this qualitative study, supported by Social Representation Theory’s procedural aspect, was conducted with 37 students who were tuberculin-positive and enrolled in an undergraduate nursing program at a public university in Belém, Pará, Brazil. Individual interviews were carried out, and the *corpus* was subjected to the software *Interface de R pour les Analyses Multidimensionnelles de Textes et de Questionnaires* 0.7/alpha 2 for lexical analysis mediated by Descending Hierarchical Classification.

**Results:**

social representations were revealed through participants’ personal experiences and the reflections they undertook after the positive result, aiming to understand how they may have been exposed to the bacillus and become infected.

**Final Considerations:**

to protect students in their personal and academic daily lives, biosecurity measures require collaborative partnerships between the health and education sectors, but it is also necessary to strengthen Primary Health Care actions.

## RESUMEN

**Objetivos:**

analizar las representaciones sociales de estudiantes de enfermería respecto a la infección tuberculosa latente.

**Métodos:**

estudio cualitativo, fundamentado en el aspecto procesual de la Teoría de las Representaciones Sociales. Se realizó con 37 estudiantes con diagnóstico positivo de tuberculosis, matriculados en un programa de pregrado de enfermería en una universidad pública de Belém, Pará, Brasil. Se realizaron entrevistas individuales y el *corpus* se sometió al *software Interface de R pour les Analyses Multidimensionnelles de Textes et de Questionnaires* 0.7/*alpha* 2 para el análisis léxico mediado por la Clasificación Jerárquica Descendente.

**Resultados:**

las representaciones sociales se revelaron a través de las experiencias personales de los participantes y las reflexiones que realizaron después del resultado positivo, con el objetivo de comprender cómo pudieron haber estado expuestos al bacilo y haberse infectado.

**Consideraciones Finales:**

para proteger a los estudiantes en su vida diaria, tanto personal como académica, las medidas de bioseguridad requieren alianzas de colaboración entre los sectores de salud y educación, pero también es necesario reforzar las acciones de la Atención Primaria de Salud.

## INTRODUCTION

Caused by *Mycobacterium tuberculosis*, tuberculosis (TB) is a chronic infectious disease transmitted through the airways, when bacilli expelled by sneezing, talking, or coughing from infected individuals are inhaled^(1)^. In addition to TB presenting in pulmonary and extrapulmonary forms, which affect the lungs and other organs or tissues, it is important to note that there is a possibility of infection by *Mycobacterium tuberculosis* without necessarily developing the disease, a condition known as latent tuberculosis infection (LTBI), in which there are no signs or symptoms^(2)^.

In this condition, immunocompetent individuals act as reservoirs, as they live with bacilli for years or throughout their lives without becoming ill. This is possible because the immune system keeps them contained until, in some cases, due to successive exposures or alterations in defense mechanisms, the bacilli are reactivated. It is known that such alterations are related to age (younger than two and older than 60 years) or result from the continuous use of immunosuppressive medications or conditions such as malnutrition, diabetes mellitus, and infection with the human immunodeficiency virus^(3)^. It is estimated that 1/4 of the world’s population is infected^(4)^ and that 10% of this contingent will become ill with TB: 5% in the first two years after infection and 5% throughout their lives^(1,3)^.

The tuberculin skin test is the most widely used test for diagnosing LTBI in Brazil. It is based on a cutaneous hypersensitivity reaction to tuberculin, applied in a small volume (0.1 milliliter) intradermally, preferably on the anterior aspect of the left forearm. After a minimum interval of 48 and a maximum of 96 hours, the reading and interpretation should be performed with a ruler to measure the largest transverse diameter of the indurated area, considering an individual with an induration equal to or greater than 5 millimeters (mm) as positive or infected^(5)^.

Among the groups most exposed to the bacillus, undergraduate nursing students stand out, due to their work in healthcare services and other community settings, assisting people with varied clinical conditions, such as those living with TB and their contacts, whose follow-up occurs especially in Primary Health Care (PHC)^(6)^. Proof of this is that other studies have already demonstrated a significant prevalence of LTBI in this population^(6,7)^.

Upon discovering they tested positive, it is opportune to recognize that subjective aspects surround LTBI in students’ daily lives, since this diagnosis can influence thoughts and actions that will allow them to adapt to the new reality. Therefore, LTBI’s psychosocial character is evident, causing students to produce meanings that will guide behaviors based on how they represent, or can represent, the fact of living with such a condition.

Since this implies the need to understand it beyond clinical-epidemiological aspects, it is understood that using the Social Representation Theory (SRT) can support timely reflections. Created by Serge Moscovici in the field of social psychology, this theory consists of the investigation of a particular form of knowledge called social representations (SRs), which guides communication and behavior^(8)^. Produced, modified and shared in time and space, through available communication resources^(9)^, SRs align with TB’s socio-historical nature^(10)^, which provides the background for understanding the infection.

Engaging with this information, research investigating the knowledge, attitudes, and practices of health science students (medicine, pharmacy, and public health) affiliated with the University of Liberia found a lower rate of correct answers, indicating a considerable gap in knowledge related to LTBI. The study considered the possibility that this gap might be caused by factors such as inaccurate reports of real-life cases, students’ likely clinical exposure to the bacillus during practical activities in healthcare services, and their contact with socially determined beliefs, norms, and values, which often induced thoughts that surpassed technical-scientific knowledge, influencing attitudes and practices that deviated from the rigors of that knowledge^(11)^.

Strategies aimed at identifying gaps of this nature, in addition to cultural and socioeconomic strategies, should be adopted to facilitate LTBI control^(12)^, especially among groups that express greater susceptibility to contagion, such as nursing students. In light of SRT and its applicability in this context, it is understood that the psychosocial perspective favors the unveiling of phenomena that dynamically encompass knowledge derived from common sense and knowledge from the scientific field called “consensual universe” and “reified universe”. It is known that human groups have cognitive abilities and social needs to use them in their daily relationships, when weaving conversations on various topics and acting in response to them^(13)^.

Qualitative research conducted in Cape Town, South Africa, affirmed that, due to the increasing emphasis on treating LTBI in high-risk groups, there is an urgent need to understand these groups’ knowledge and practices regarding the topic, aiming to create and implement coping guidelines that consider sociocultural aspects^(14)^.

Despite the extensive global scientific production on LTBI, developed by researchers from various areas^(15)^, these data confirm that it is still necessary to invest, in a timely manner, in research that unveils the SRs linked to the phenomenon, especially in the social and symbolic contexts of nursing students, supporting this study’s relevance.

This argument aims to understand their subjectivities, since meanings are related to students’ academic and personal experiences, and reflective capacity, who engage with common sense and scientific knowledge on topics that circulate in everyday life, reinforcing SRT’s applicability in the experiential context of these people.

## OBJECTIVES

To analyze nursing students’ social representations regarding latent tuberculosis infection.

## METHODS

### Ethical aspects

The study complied with Resolution 466/2012 of the Brazilian National Health Council, and was approved by the Research Ethics Committee of the university where it took place, being authorized by the same institution. Participant acceptance was obtained upon signing the Informed Consent Form (ICF). In order to protect the confidentiality of their identities, alphanumeric codes were established with the following composition: the letter S, alluding to the word “student”, followed by a Roman numeral indicating the year of the course, a hyphen, and an Arabic numeral indicating students’ ordinal position in the sequence of interviews.

### Study design and theoretical-methodological framework

This is a descriptive and qualitative study, supported by the SRT’s procedural aspect^(13)^. Writing was guided by the COnsolidated criteria for REporting Qualitative research (COREQ) checklist recommendations, considering items that comprise its three domains intrinsic to the team, the study design, and the set formed by analysis and results^(16)^. This theory explains that two cognitive processes, based on social memory and called anchoring and objectification, make it possible to construct SRs^(8)^.

Aiming to familiarize psychosocial objects initially perceived as strange and distant from reality, anchoring allows them to be named and allocated into known mental categories based on daily experiences, establishing social values according to human groups’ norms or precepts. In turn, objectification assigns images or symbols, transferring the elaborations of the psychic plane to the physical plane in an attempt to materialize them and thus bring them closer to reality. These processes occur through a dialogical relationship between the conceptual nature of the object, which gives it meaning, and its figurative nature, which assigns it an icon^(8)^.

### Study setting and data source

This study was conducted as part of an undergraduate nursing course at a public university in Belém, the capital of the state of Pará, Brazil, within the geographical and social context of the so-called Metropolitan Region of Belém, comprised of seven other municipalities that work together to strengthen the organization, planning, and execution of public policies^(17)^.

The course lasts five years and has a workload of 5,000 hours. It is structured in five years and ten semester chunks, with each year consisting of two chunks, corresponding to the 1^st^ and 2^nd^ semesters. The teaching staff is composed of nurses and professionals from other areas, holding specialist, master’s, or doctoral degrees and with permanent or temporary affiliations. Curricular components are distributed across 11 departments. The course coordination is the responsibility of a nurse professor who conducts activities in the capital and in the countryside of the state^(18)^.

Thirty-seven students enrolled in the five years of the course participated, all of whom were tuberculin-positive (defined as those with induration equal to or greater than 5 mm). Induration was determined through testing conducted as part of a multicenter study led by the authors’ institution, in partnership with three other public universities located in the capital cities of the states of Amazonas, Piauí, and Rio de Janeiro, aiming to determine the prevalence of LTBI in this population.

In the municipality of Belém, testing took place between December 2022 and February 2023, reaching 269 participants and 74 who tested positive (27.51%). An initial plan was to include half of students who tested positive, understanding that this sample would be numerically representative. However, during the research, the authors chose to also consider theoretical saturation, as it constitutes an important element of the qualitative approach, especially within a specific theoretical-methodological framework. Thus, saturation was identified when the data proved satisfactory for achieving the objective and additions would likely not alter the understanding of the representational phenomenon^(19)^. As statements were obtained, careful checks were made to determine if saturation had been reached.

Students who tested positive that could not be located and those that indicated impediments to scheduling an interview were excluded, with no withdrawals.

### Data production

In order to obtain authorization, the first author visited the university to present the project to the course coordinator, at which time they agreed to the use of a room to conduct the individual interviews, which took place between March and July 2023. The training processes developed in a research group linked to the authors’ institution and the orientation meetings of the master’s course in nursing, in which the author was enrolled, qualified him to conduct the interviews.

With the list of participants, containing names, phone numbers, and information about the tuberculin skin test, provided by researchers from the multicenter study, and with students’ due authorization, the author contacted them by phone or messaging app, briefly explaining the research and inviting them to participate. Those who accepted were instructed to come to a reserved room on the scheduled day and time, where only the author and the participant were present, respecting their privacy. As strategies to avoid COVID-19 in an enclosed environment, 70% alcohol gel was made available, and participants were informed of the possibility of wearing a mask.

Initially, the objectives, procedures, risks, and benefits were detailed in the ICF, clarifying any doubts to obtain formal acceptance. The interviews were guided by a semi-structured script, constructed by the first two authors and submitted to the assessment of four researchers in the field of nursing, with solid scientific production and experience in guiding undergraduate and graduate students. Among them, one developed studies on SRs, and all were experts in topics related to communicable diseases, higher education, and public health. After assessment, two endorsed the instrument, and the others offered suggestions to strengthen its content, which were considered, and appropriate aspects were accepted. Since it was deemed suitable, it was decided not to submit it to a pre-test or pilot test.

In the first axis of the script, the sociodemographic and academic profile was investigated through objective questions that encompassed variables of group belonging, understanding that subjects speak from the places they occupy in society, resulting in the contextualization of SRs^(11)^. These variables were age, sex, school year, race/skin color, religion, marital status, number of children, perception of scholarship, occupation, family income, municipality of residence, number of people with whom they lived, and, additionally, induration size. In the second axis, the representational phenomenon was investigated with subjective questions that allowed the identification of experiences and reflections on the subject. The interviews lasted from 20 to 50 minutes and were recorded in MP3 format using digital equipment.

Since the author was part of the multicenter study team and operationalized testing, students knew him and were aware that research with participants who tested positive was a requirement for completing the master’s course. It should be noted that the content of the interviews was not shared with them for their endorsement, as this would compromise the spontaneity of the statements, valued as a fundamental element for the SRT. Given the completeness of results, the interviews were not repeated and no other production techniques were adopted.

### Data organization and analysis

Sociodemographic and academic profile data were stored in the Microsoft Office Excel^®^ 2013 version, created by Microsoft Corporation. This enabled descriptive statistical analysis, which identified absolute and relative values.

Responses to subjective questions were transcribed to generate a *corpus*, following the *Interface de R pour les Analyses Multidimensionnelles de Textes et de Questionnaires* (IRaMuTeQ^®^) version 0.7 (alpha 2) technical and methodological guidelines^(20)^, created by Frenchman Pierre Ratinaud, with whom lexical analysis was carried out in Portuguese, considering that it presents complete dictionaries in several languages^(21)^.

The *corpus* was organized into a single, unformatted file (.txt), with character encoding in the Unicode Transformation Format 8-bit standard. By using statistical functions of the R software and the Python programming language, IRaMuTeQ^®^ allows for the investigation of textual data with scientific rigor, such as those originating from a set of transcripts on a specific topic, as is the case with the *corpus* of this study^(20)^. Furthermore, since it is freely accessible, IRaMuTeQ^®^ has been widely used in qualitative research^(21)^, including with SRT^(22)^.

Its menu offers five modalities: lexicographical statistics; specificities and factorial correspondence analysis; Descending Hierarchical Classification (DHC); similarity analysis; and word cloud. Among these, DHC was chosen, aiming to break down the *corpus* into text segments (TSs), which composed lexical classes formed by words arranged vertically in a dendrogram, considering as representative those that obtained p<0.0001. The semantic elements of these words were identified by TSs in which they were located and which gave them meaning. Four statistical values were assigned to them: frequency of TSs containing the word in the class (Fc); total frequency of TSs containing the word in the *corpus* (Tf); percentage of TSs containing the word in the class (%) in relation to its occurrence in the *corpus*; and word’s associative strength, demonstrated by the chi-square test (X^2^), which defines its ordinal position in the class^(20)^.

Due to the organization carried out by DHC, it is often not possible to use all TSs, which is why authors acknowledge that, for a reliable analysis, the minimum utilization percentage should be 75%^(21,23)^. Data were interpreted and discussed based on evidence from the scientific literature on the subject and on anchoring and objectification processes, whose concepts were presented in the theoretical-methodological framework.

## RESULTS

Among the participants, the age ranged from 19 to 40 years, with 28 (75.68%) in the 19-23 age range and 24 (64.86%) being women. Concerning school years, seven (18.92%) were in the 1^st^ year, five (13.51%) in the 2^nd^, eight (21.62%) in the 3^rd^, ten (27.03%) in the 4^th^, and seven (18.92%) in the 5^th^. In relation to other variables, 21 (56.76%) identified as brown, 17 (45.95%) as evangelical, and 32 (86.49%) as single. Furthermore, 35 (94.59%) had no children; 22 (59.46%) did not receive scholarships; 31 (83.78%) did not have professional activities; and 23 (62.16%) declared a family income between two and three minimum monthly wages.

All resided in municipalities within the Metropolitan Region of Belém, with one to 18 people living in the same household. In this context, one participant (2.70%) stated that they lived in their own home with 13 people, and another (2.70%) with 18, including extended family members and relatives of varying degrees of kinship. However, a large portion (n=13; 35.14%) stated that they lived with only three people, among whom those in the immediate family circle stood out, mainly mother, father, and siblings. Induration size, generated by the tuberculin test, ranged from five to 20 mm, with an average of 11.70 mm, with 13 (35.14%) presenting 10 mm.

With 37 texts, IRaMuTeQ^®^ generated 1,939 TSs by disassembling DHC, resulting in the utilization of 1,686 (86.95%), considered satisfactory. Moreover, 67,619 occurrences were identified, corresponding to the total number of forms or words, of which 5,022 were distinct words and 2,367 were words with only one frequency.

**Figure 1:**
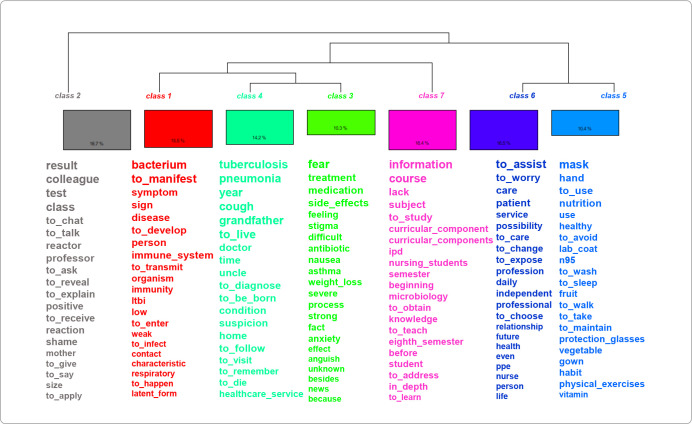
Descending Hierarchical Classification vertical dendrogram showing the logic of *corpus* partitioning, the percentages of text segments (standardized by IRaMuTeQ^®^, with one decimal place after the comma) and the initial words of lexical classes, Belém, Pará, Brazil, 2023

The *corpus* partitioning resulted in two *subcorpora*, consisting of seven lexical classes, such that, in the 1^st^ *subcorpus*, class 2 was allocated, accounting for 281 TSs (16.67%), and the other six were allocated in the 2^nd^ *subcorpus*, in this order: class 5 accounted for 175 TSs (10.38%); class 6 accounted for 278 TSs (16.49%); class 7 accounted for 277 TSs (16.43%); class 1 accounted for 262 TSs (15.54%); class 3 accounted for 174 TSs (10.32%); and class 4 accounted for 239 TSs (14.18%). Thus, classes 2 and 3 were considered the largest and smallest among the seven, respectively, as shown in the DHC vertical dendrogram, generated by IRaMuTeQ^®^, with the initial words of each class (Figure 1).

*IPD - infectious and parasitic diseases; LTBI - latent tuberculosis infection; PPE - personal protective equipment.*

These words reveal a diverse set of thoughts and social practices related to students’ SRs on the topic. For this reason, the classes were grouped into three thematic axes, constituting the results of the original research. Based on the complementarity and semantic convergence among the classes, the axes were named and organized as follows: I) “Giving meaning to LTBI and TB: knowledge, affections and memories”, with classes 1, 3, and 4; II) “Testing positive: discovering, caring for oneself and others”, with classes 2, 5, and 6; III) “Informing to better care: challenges and possibilities in higher education”, with class 7.

In order to meet the objective of this study, it was decided to highlight class 4, which constituted the 2^nd^ *subcorpus* and presented 60 representative words (p<0.0001), of which 30 (50%) were detailed in Table 1, as they were the most representative. This choice is justified both by the large volume of data from the original research, which makes its adequate presentation in just one manuscript unfeasible, and by the completeness and relevance of results of class 4, which allow it to be explored separately and with the necessary theoretical and methodological depth.

**Table 1:** Lexical detailing of class 4, Belém, Pará, Brazil, 2023

Nº	Words	Fc	Ft	%	X^2^
1	Tuberculosis	133	510	26,08	85,15
2	Pneumonia	18	22	81,82	83,83
3	Year	16	19	84,21	77,47
4	Cough	23	38	60,53	68,65
5	Grandfather	22	37	59,46	63,76
6	To_live	17	27	62,96	53,68
7	Doctor	20	36	55,56	51,77
8	Time	17	29	58,62	47,91
9	Uncle	17	30	56,67	45,33
10	To_diagnose	13	20	65,00	42,97
11	To_be_born	7	7	100,00	42,56
12	Condition	8	9	88,89	41,52
13	Suspicion	8	9	88,89	41,52
14	Home	25	60	41,67	38,65
15	To_follow	10	14	71,43	38,04
16	To_visit	6	6	100,00	36,46
17	To_remember	33	95	34,74	34,98
18	To_die	14	26	53,85	34,16
19	Healthcare_service	5	5	100,00	30,36
20	Father_in_law	6	7	85,71	29,57
21	My_God	9	14	64,29	29,14
22	To_catch	24	66	36,36	27,80
23	Father	22	60	36,67	25,87
24	There	11	21	52,38	25,51
25	Death	4	4	100,00	24,28
26	Neighborhood	4	4	100,00	24,28
27	Reclusive	4	4	100,00	24,28
28	Child	8	13	61,54	24,16
29	To_treat	16	40	40,00	22,46
30	Family	25	78	32,05	21,48

*Fc - frequency of text segments containing the word in the class; Tf - total frequency of text segments containing the word in the corpus; % - percentage of text segments containing the word in the class in relation to its occurrence in the corpus; X^2^ - chi-square.*

This class presented TSs that relate to participants’ non-academic experiences in engaging with the topic and the reflections they undertook after the positive result, in order to better understand in which possible occasions they were exposed to the bacillus, causing LTBI.

Thus, various experiences were revealed involving close people, such as friends and relatives, or people with whom the relationship was distant and sporadic. In this context, family experiences of illness from TB or other respiratory diseases, which occurred recently or several years ago, with grandparents, parents, and uncles, among other relatives, stood out.

This aspect is amply illustrated by the words with the greatest force (“tuberculosis” (X^2^=85.15) and “pneumonia” (X^2^=83.83)), associated with words like “cough” (X^2^=68.65), “grandfather” (X^2^=63.76), “doctor” (X^2^=51.77), “time” (X^2^=47.91), “uncle” (X^2^=45.33), “to diagnose” (X^2^=42.97), “suspicion” (X^2^=41.52), “to die” (X^2^=34.16), “father-in-law” (X^2^=29.57), “father” (X^2^=25.87) and “reclusive” (X^2^=24.28), as demonstrated in the following excerpts:

*My mother-in-law was hospitalized with suspected TB and had a prolonged dry cough. When we sought medical attention for her admission, because she had a history of pneumonia, we asked the doctor if it wouldn’t be a good idea to test for TB. She was hospitalized for that reason and, thank God, he ruled out TB, but she remained hospitalized again with pneumonia.* (SIII-11)

*My uncle contracted TB in 2022, became very ill, and had to undergo lung surgery. At the time, I was in contact with him because he used to come to my house. We weren’t in contact during the most severe stage of the disease, because afterward* [when his condition worsened] *he became reclusive. Before that, even he didn’t know he was sick, because he’d always been like that - someone who had illnesses and didn’t worry about them. I don’t know if my father caught it from my uncle, because my father also has TB, was diagnosed recently, and is undergoing treatment.* (SI-26)

*In my family, we’ve discussed how my grandmother had pneumonia, but it’s not known if it was just pneumonia because, back then, there was no screening. She worked selling açaí, didn’t have access to a doctor, and when she finally had a CT scan, she was already elderly and had a chronic cough. When she had a chest X-ray, it was only after a chest X-ray* [referring to chest x-ray]*, There was already calcification at the base of the lung, but she didn’t die from that. It was only observed, and my mother associated it with the supposed case of pneumonia that my grandmother had and treated with home remedies.* (SIV-33)

Among those who reported having discussed TB, or having developed another form of contact with the topic through audiovisual media (such as television), before entering the course or outside the academic environment, an association of the disease with historical events or movements was observed, such as Romanticism, an artistic and cultural movement dating from the 18^th^ and 19^th^ centuries, and the Second World War, which occurred in the 20^th^ century.

Influenced by their high school experiences, some participants anchored the occurrence of TB to conditions of deprivation and suffering, as these weaken the body, predisposing it to illness. This idea was objectified by the use of expressions such as “evil of the century” and “Nazi Germany”, which refer to historically documented and perpetuated moments of crisis, configuring an image projection of such conditions:

*Another memory I have is from literature class in school, where we had Romanticism, the second generation, and the evil of the century, because it was very common for writers to die of TB. Their suffering influenced their writing, how they treated the story, how they saw the world within the narrative. This sparked my curiosity about the disease.* (SI-10)

*Some films reminded me of Romanticism, where people died from TB, which was the evil of the century. People would catch* [the disease]*, go to the mountains to seek fresh air, trying to get better, but many would die. I remember seeing this in films and also studying literature about the Romantic Period in high school.* (SII-12)

*There were many cases of TB during the Nazi Germany era, in the concentration camps, precisely because people had low resistance* [immunity]*, were malnourished, did not live in decent conditions, and therefore were more likely to get sick.* (SI-26)

All participants acknowledged that they had been exposed to the bacillus at some point, either inside or outside the academic environment, although they could not pinpoint when the infection occurred. However, in an attempt to find a link to the positive result, participants recalled situations that, in their view, may have caused or contributed to LTBI, anchoring it in three main factors that occurred jointly or separately: 1) contact with people who had TB; 2) contact with people who had other respiratory diseases or were suspected of having TB; 3) exposure to environments with high biological risk, such as health units and hospitals, to carry out activities during the course, but also, in the case of some participants, to receive medical care, mainly during childhood or adolescence.

This reflective attitude is denoted by the word “to remember” (X^2^=34.98), associated with words like “year” (X^2^=77.47), “to live” (X^2^=53.68), “home” (X^2^=38.65), “healthcare service” (X^2^=30.36), “child” (X^2^=24.16), and “family” (X^2^=21.48), referring to the locations and circumstances in which exposure and contagion possibly occurred:

*My great-grandmother, who passed away many years ago, had TB, and I cared for her for three years during the last years of her life. I cared for her after her treatment! Before the illness, she already lived in my house, so I believe I came into contact with the bacteria because of her.* (SII-4)

*In practical classes, we sometimes came into contact with people who were suspected of being ill, but we never had confirmation. Therefore, I don’t know if I contracted LTBI while interning in healthcare services or if I acquired it somewhere else, like on public transport. When I was younger, I had a lot of allergies, so I went to the doctor a lot. In addition, I went to the orthopedist because I always had back problems.* (SIII-7)

*When I was a child, I practically lived in the hospital* [seeking medical care]*. I only stopped going to the hospital when I was about 12 years old. Since I was born, I’ve had many bouts of pneumonia, and I was constantly hospitalized. Because I’ve always had respiratory problems, perhaps because of that, being in the hospital, I may have become susceptible* [infected]*, or even on some random street, I may have come into contact with the bacteria.* (SI-13)

*When I learned that my grandmother was suspected of having TB, I thought, “My God, I believe more people in the family may be infected, including me, since I had contact with my father”* [who contracted the disease]*. Because I didn’t show any symptoms, I didn’t immediately seek testing for myself.* (SIV-29)

## DISCUSSION

Based on social memory, the results present the non-academic experiences surrounding the topic and participants’ reflective attitude in linking the positive result to past events. It is understood that the recollection of experiences and such a reflective attitude contribute to normalizing this unusual event, i.e., testing positive to the tuberculin test and living with LTBI.

In the field of representational phenomena, naturalization occurs to establish subjects’ sociocultural identity with the object represented, referring to anchoring and objectification processes^(24)^, which do not necessarily unfold in that order, as they are dynamic and interdependent, occurring simultaneously^(25)^.

Once an object is anchored, i.e., integrated into a pre-existing system of knowledge, objectification causes it to be perceived as real by individuals and by their community^(9)^. Thus, to fulfill its purpose, anchoring unfolds in three stages: meaning attribution, through a set of pre-existing meanings; knowledge instrumentalization, which corresponds to functionality; and iconic transformation, the moment when SRs become familiar. In turn, objectification expresses three aspects: selection and decontextualization, in which certain elements are highlighted from the information network; figurative or imaginative core construction; and naturalization, identifying the object in reality^(26)^.

Family members’ illness due to TB or other respiratory conditions became evident, simultaneously bringing to the surface affections and memories surrounding the disease as strong markers of the SRs, given the cognitive access to information alluding to the loved ones’ illness.

In dialogue with this result, studies analyzed SRs of *quilombola* women from the Amazon region of Pará regarding healthcare, presenting reports of illness among family members and other community members. They highlighted how the women cared for these people and the explanations that justified the actions undertaken^(27,28)^. The stereotypical figure of a “supermom” was constructed, representing the conceptual idea and visual projection of a woman with diverse attributes and qualities, who cares for the home, family, and community with affection and dedication^(27)^.

Influenced by the knowledge acquired in high school and the experiences they built in that context, participants verbalized the metaphorical expression “disease of the century” and the term “Nazi Germany” to refer to TB, its causes, and its multifaceted effects, objectifying it as a historical entity and a social ill. In this understanding, the disease is a product of subhuman living conditions, especially those linked to poverty or low income, and inadequate social support situations, as reiterated by several national^(29,30)^ and international^(31,32)^ studies.

However, it is worth highlighting that Brazil has several government social support and income transfer programs, even with operational obstacles related, among other factors, to the low level of knowledge and limitations imposed by health and social assistance professionals’ administrative governance, as sometimes occurs among those working in PHC^(29,33)^. Some of these programs include the Continuous Benefit Payment and the *Bolsa Família* Program (Family Allowance Program), in addition to other initiatives, which include, for instance, disability retirement and retirement for low-income individuals, tax exemption on income related to retirement or pensions, sick pay, and financial assistance for food and transportation^(34)^.

Although not specifically aimed at people with TB, these programs are directed at human groups facing challenging living or health conditions, which necessarily includes TB illness. The goal is to mitigate the socioeconomic impact on individuals’ and their family’s budget, encourage continued treatment to achieve a cure, and improve quality of life^(30)^.

Reaffirming the need to promote adequate social support in the context of TB, the Ministry of Health has prepared a guide to clarify various aspects, such as the protection mechanisms that exist in the national territory and how to expand them, as well as the possibilities for integrating the Brazilian Health System (In Portuguese, *Sistema Único de Saúde* - SUS) and the Unified Social Assistance System (In Portuguese, *Sistema Único de Assistência Social* - SUAS) actions, and the importance of civil society strengthening this integration^(34)^. Therefore, the Ministry of Citizenship and the Ministry of Health jointly published Operational Instruction 1, dated September 26, 2019, reinforcing the coordination between the SUS and SUAS to enable a collective response to TB^(35)^.

Given that men construct their thoughts based on the web of knowledge they have access to^(8)^, traditional forms of thought contribute to anchoring new knowledge (in this case, LTBI among those who live with it) in socially elaborated and shared categories, allowing subjects to understand it^(13,36)^, which is why it is understood that TB constituted the backdrop in which students constructed and expressed their SRs. In view of this, anchoring situates the role of social memory in the symbolic construction of reality so that men understand it and act upon it. Thus, science interacts with common sense, without this interaction necessarily provoking oppositions in relation to traditional forms of thought^(36)^.

In relation to TB’s psychosocial construction as a historical entity, it is known that the 19^th^ century represented the most critical moment for the disease in the world and in Brazil, a country where the number of deaths caused by it was countless, justifying its designation as the “disease of the century”. Since, at that time, individual and collective protection measures were incipient and very limited, TB became an even greater public health problem among those with habits considered unruly, as they were more vulnerable to illness due to their bohemian lifestyle, permeated by excesses and vices, as well as the desire to “die of love” in the face of unrequited romantic feelings^(37)^.

As evidenced by some participants, this was the case for Byronic poets, since almost all those of the second phase of Romanticism in Brazil died young as a result of TB. Examples of this are Álvares de Azevedo (1831-1852), who died at age 21, Casimiro de Abreu (1839-1860), who also died at age 21, and Castro Alves (1847-1871), who died at age 24^(37)^. TB’s anchoring to World War II is also supported by the literature, as the disease was understood by soldiers as a war risk, from which they needed to defend themselves and their homeland^(38)^.

Recognizing that they had previous contact with the bacillus and therefore live with LTBI, students reflected on the possibility of having been exposed in situations of contact with people affected by TB or other respiratory diseases, or even in high-risk environments, especially in healthcare services, where they carried out activities during the course or sought some type of care during childhood or adolescence. This epidemiological notion of risk and exposure is endorsed by the Ministry of Health^(1,3)^ and through different studies^(2,6,7)^.

Although adequate knowledge of the subject is fundamental in this audience’s personal and academic lives, envisioning future professional practice, research conducted with 60 nursing students from a federal university located in the municipality of Três Lagoas, Mato Grosso do Sul, identified limited knowledge among students with less university study time and who had no prior contact with the subject, contrasting with the better-structured knowledge among those who obtained information in healthcare services. This indicated that teaching-learning strategies need to be strengthened, rethought, or restructured in nursing education, providing opportunities for the timely sharing and acquisition of information on such a relevant topic^(39)^.

This assessment takes into account TB’s current epidemiological scenario in Brazil^(40)^ and in the world^(4)^, reinforced by the high prevalence of LTBI and by several structural factors that, together, maintain the disease as a serious public health problem^(4,40-43)^. It should also not be forgotten that the topic is still shrouded in fears, stigmas, and prejudices, crystallized over time and manifested in human groups’ daily lives, including during nurses’ professional practice in PHC^(10)^.

Supporting this reflection, research conducted in the municipality of Bolgatanga, in the upper eastern region of Ghana, involved three focus groups totaling 19 participants, of whom 13 lived with TB and six did not. The research revealed a strong manifestation of community stigma against those living with TB, as people without the disease were afraid to approach them, reinforcing conditions of isolation and social distancing^(44)^.

This hinders effective control through actions that must be carried out with individuals, their families and their communities, which includes investigating, treating and monitoring cases of LTBI among contacts^(1,3)^, but also points out that health education strategies need to be rationally adopted. This consideration is made with the aim of transforming knowledge and practices in the community context beyond teaching-learning strategies that make it possible to strengthen both academic training in higher education and professional training in the daily routine of healthcare services.

### Study limitations

Given that the study was conducted in an undergraduate nursing program at a public higher education institution in the municipality of Belém, thus configuring a specific reality, it is understood that the data were determined by aspects that contextualize SRs, such as educational, geographic, and sociocultural factors. This resulted in the impossibility of considering that the results, in their entirety, have the potential to converge with the personal and academic realities faced by nursing students who experience the course in other public or private higher education institutions in the state of Pará, in other states of the North region, or in the rest of the Brazilian territory.

However, at least partially, results may converge with these individuals’ realities, since the fact that they are students brings them closer, helping them to better understand the topic and share it through experiences inside and outside the classroom.

### Contributions to nursing, health, or public policy

Based on the results and their discussions, public health authorities and academic administrators can reflect on the need to propose, maintain, or restructure measures that improve biosafety conditions in environments where nursing students carry out activities both inside and outside the university. In this context, healthcare services that provide care to people with TB and their contacts stand out, with the aim of preventing the occurrence of long-term TB infection among students.

In light of the reflections presented here, spaces for discussion can be fostered within the academic community and among healthcare professionals, aiming to clarify aspects related to the topic and democratize its timely sharing. This supports the use of SRs as forms of knowledge that enable a better understanding of the reality in which one is immersed, in order to act within it, in a constant movement of cognitive and perceptual feedback, which reaffirms the dynamic and pragmatic nature of such representations.

## FINAL CONSIDERATIONS

This study revealed nursing students’ experiences and reflections on LTBI, expressed through their SRs. In this context, several non-academic experiences were reported, especially those involving illness from TB or other respiratory conditions affecting family members, as well as everyday school situations in high school through which they came into contact with the topic. The reflections undertaken after a positive test result were particularly noteworthy, aiming to better understand the circumstances in which they were possibly exposed to the bacillus and became infected, establishing a link to justify this condition.

Aiming to protect students, both in their personal and academic lives, biosecurity measures require collaborative partnerships between the health and education sectors, integrating their representatives to achieve this goal. However, the results demonstrate that it is also necessary to strengthen care actions, especially in PHC so that patients are identified, treated, and monitored in a timely manner, breaking the chain of transmission to prevent a greater number of infected individuals and new cases of illness.

By demonstrating the need for integration between these sectors, it is hoped that the results will mobilize students and faculty-researchers in nursing and related fields, such as education, psychology, and public health, to invest in the topic so that other studies can be carried out, encompassing scenarios and objects inherent to care, healthcare service management, teaching, research, and social leaders’ political participation in instances of power and governance. The SRT can underpin the conception, development, and interpretation of these studies, providing an opportunity to overcome possible theoretical and methodological weaknesses.

Thus, it will be possible to understand aspects that have not yet been investigated or are poorly understood, as well as to broaden the dissemination of the topic through publications in scientific journals and other reliable sources, and through the culturally appropriate use of these materials by qualified professionals. This aims to transform, to some degree, the reality of the human groups they assist, especially in the face of the challenges posed by LTBI.

## Data Availability

The research data are available only upon request.
