# Supplementary material for: Latent tuberculosis infection among undergraduate nursing students: a study of social representations
Source: Rev Bras Enferm. 2026 Jul 24;79:e20250282. doi: 10.1590/0034-7167-2025-0282 (PMC13403634; doi:10.1590/0034-7167-2025-0282)
Supplement: Translation

# Translation: Infecção latente da tuberculose entre estudantes de graduação em enfermagem: estudo de representações sociais

**DOI:** 10.1590/0034-7167-2025-0282pt

**Authors:** Erlon Gabriel Rego de Andrade, Ivaneide Leal Ataide Rodrigues, Laura Maria Vidal Nogueira, Alexandre Aguiar Pereira, Eliza Paixão da Silva, Adriely Alciany Miranda dos Santos

**Affiliations:** I Universidade do Estado do Pará. Belém, Pará, Brasil

**Keywords:** Tuberculose Latente, Estudantes de Enfermagem, Representação Social, Psicologia Social, Saúde Pública.

## Abstract

**Objetivos:**

analisar as representações sociais de estudantes de enfermagem sobre a infecção latente da tuberculose.

**Métodos:**

estudo qualitativo, subsidiado pela vertente processual da Teoria das Representações Sociais. Ocorreu com 37 estudantes reatores à prova tuberculínica, matriculados no curso de graduação em enfermagem de universidade pública em Belém, Pará, Brasil. Foram realizadas entrevistas individuais, submetendo-se o *corpus* ao *software Interface de R pour les Analyses Multidimensionnelles de Textes et de Questionnaires* 0.7/*alpha* 2 para análise lexical mediada pela Classificação Hierárquica Descendente.

**Resultados:**

as representações sociais foram desveladas por meio de experiências pessoais dos participantes e das reflexões que empreenderam após o resultado reator, visando compreender como possivelmente se expuseram ao bacilo e foram contaminados.

**Considerações Finais:**

para proteger os estudantes em seu cotidiano pessoal e acadêmico, ações de biossegurança requerem parcerias colaborativas entre os setores de saúde e educação, mas também é necessário fortalecer as ações da Atenção Primária à Saúde.

## INTRODUÇÃO

Causada pelo *Mycobacterium tuberculosis*, a tuberculose (TB) é uma doença infectocontagiosa crônica transmitida pelas vias áreas, quando se inalam bacilos expelidos por espirro, fala ou tosse de pessoas doentes^(1)^. Além de a TB se apresentar nas formas pulmonar e extrapulmonar, que acometem os pulmões e outros órgãos ou tecidos, cabe ressaltar que existe a possibilidade de infecção pelo *Mycobacterium tuberculosis* sem necessariamente desenvolver a doença, condição conhecida como infecção latente da tuberculose (ILTB), na qual inexistem sinais e sintomas^(2)^.

Nessa condição, pessoas imunocompetentes funcionam como reservatórios, pois vivem com bacilos por anos ou durante toda a vida, sem adoecerem. Isso é possível porque o sistema imunológico os mantém retidos, até que, em alguns casos, por sucessivas exposições ou alterações nos mecanismos de defesa, os bacilos sejam reativados. Sabe-se que tais alterações se relacionam à idade (menor que dois e maior que 60 anos) ou resultam do uso contínuo de medicamentos imunossupressores ou de condições como desnutrição, diabetes *mellitus* e infecção por vírus da imunodeficiência humana (HIV)^(3)^. Estima-se que 1/4 da população mundial esteja infectada^(4)^ e que 10% desse contingente adoecerá por TB: 5% nos dois primeiros anos após a infecção e 5% ao longo da vida^(1,3)^.

A prova tuberculínica é o teste mais utilizado para diagnosticar ILTB no território brasileiro. Fundamenta-se em reação de hipersensibilidade cutânea à tuberculina, aplicada em pequeno volume (0,1 mililitro), por via intradérmica, preferencialmente na face anterior do antebraço esquerdo. Após intervalo mínimo de 48 e máximo de 96 horas, a leitura e a interpretação devem ser realizadas com régua para aferir o maior diâmetro transverso da área endurada, considerando-se reator ou infectado o indivíduo que apresentar enduração igual ou superior a 5 milímetros (mm)^(5)^.

Entre os grupos mais expostos ao bacilo, destacam-se os estudantes de graduação em enfermagem, por desenvolverem atividades em serviços de saúde e outros ambientes comunitários, atendendo pessoas com condições clínicas variadas, a exemplo das que vivem com TB e de seus contatos, cujo acompanhamento ocorre, especialmente, na Atenção Primária à Saúde (APS)^(6)^. Prova disso é que outros estudos já demonstraram importante prevalência de ILTB nesse público^(6,7)^.

Ao se descobrir reator, é oportuno reconhecer que aspectos subjetivos circundam a ILTB no cotidiano dos estudantes, uma vez que esse diagnóstico pode influenciar pensamentos e ações que permitirão adaptar-se à nova realidade. Portanto, evidencia-se o caráter psicossocial da ILTB, fazendo com que os estudantes produzam sentidos que guiarão comportamentos a partir do modo como representam ou podem representar o fato de viver com tal condição.

Como isso implica a necessidade de compreendê-la para além dos aspectos clínico-epidemiológicos, entende-se que o uso da Teoria das Representações Sociais (TRS) pode subsidiar reflexões oportunas. Criada por Serge Moscovici no campo da psicologia social, essa teoria consiste na investigação de uma forma de conhecimento particular, chamada de representações sociais (RS), que norteia comunicações e comportamentos^(8)^. Produzidas, modificadas e compartilhadas no tempo e no espaço, mediante os recursos comunicacionais disponíveis^(9)^, as RS coadunam com a natureza sócio-histórica da TB^(10)^, que engendra o pano de fundo para que se compreenda a infecção.

Dialogando com essas informações, pesquisa que investigou conhecimentos, atitudes e práticas de estudantes de ciências da saúde (medicina, farmácia e saúde pública), vinculados à Universidade da Libéria, constatou menor taxa de acertos, apontando lacuna considerável nos conhecimentos relacionados à ILTB. Refletiu-se sobre a possibilidade de tal lacuna ser motivada por aspectos como testemunhos equivocados de casos da vida real, provável exposição clínica dos estudantes ao bacilo durante atividades práticas em serviços de saúde, e contato deles com crenças, normas e valores socialmente determinados, os quais induziam pensamentos que, muitas vezes, superavam o conhecimento técnico-científico, influenciando atitudes e práticas que destoavam dos rigores desse conhecimento^(11)^.

Estratégias direcionadas à identificação de lacunas dessa natureza, além de estratégias culturais e socioeconômicas, devem ser adotadas para facilitar o controle da ILTB^(12)^, sobretudo entre grupos que expressam maior suscetibilidade ao contágio, a exemplo dos estudantes de enfermagem. À luz da TRS e de sua aplicabilidade nesse contexto, entende-se que a perspectiva psicossocial favorece o desvelamento de fenômenos que abarcam, de forma dinâmica, saberes que advêm do senso comum e saberes do campo científico, denominados “universo consensual” e “universo reificado”. Sabe-se que os grupos humanos têm habilidades cognitivas e necessidades sociais para utilizá-los em suas relações cotidianas, ao tecerem conversações sobre diversos temas e agirem diante deles^(13)^.

Pesquisa qualitativa realizada na Cidade do Cabo, África do Sul, afirmou que, devido à ênfase crescente ao tratamento da ILTB em grupos de alto risco, há necessidade urgente de compreender saberes e práticas desses grupos acerca do tema, visando criar e implementar diretrizes de enfrentamento que considerem os aspectos socioculturais^(14)^.

Apesar da extensa produção científica global sobre a ILTB, desenvolvida por pesquisadores de diversas áreas^(15)^, esses dados ratificam que ainda é necessário investir, de maneira oportuna, em pesquisas que desvelem as RS ligadas ao fenômeno, principalmente nos contextos social e simbólico de estudantes de enfermagem, corroborando a relevância deste estudo.

Essa argumentação visa compreender suas subjetividades, pois os sentidos guardam relação com as experiências acadêmico-pessoais e a capacidade reflexiva dos estudantes, os quais dialogam com o senso comum e o conhecimento científico sobre temas que circulam no cotidiano, reforçando a aplicabilidade da TRS no contexto vivencial dessas pessoas.

## OBJETIVOS

Analisar as representações sociais de estudantes de enfermagem sobre a infecção latente da tuberculose.

## MÉTODOS

### Aspectos éticos

O estudo obedeceu à Resolução nº 466/2012 do Conselho Nacional de Saúde, sendo aprovado por Comitê de Ética em Pesquisa da universidade onde foi realizado, obtendo-se, também, a autorização institucional da mesma universidade. Obteve-se o aceite dos participantes a partir da assinatura do Termo de Consentimento Livre e Esclarecido (TCLE). Visando resguardar o sigilo de suas identidades, códigos alfanuméricos foram estabelecidos com a seguinte composição: letra E, alusiva à palavra “estudante”, seguida por algarismo romano, que indicou a série do curso, hífen, e algarismo arábico, que indicou a posição ordinal do estudante na sequência de entrevistas.

### Tipo de estudo e referencial teórico-metodológico

Estudo descritivo, com abordagem qualitativa, subsidiado pela vertente processual da TRS^(13)^. Sua redação foi guiada pelas recomendações do *checklist COnsolidated criteria for REporting Qualitative research* (COREQ), considerando os itens que compõem seus três domínios, intrínsecos à equipe, ao desenho do estudo e ao conjunto formado por análise e resultados^(16)^. Essa teoria explica que dois processos cognitivos, baseados na memória social e chamados de ancoragem e objetivação, possibilitam construir as RS^(8)^.

Visando familiarizar objetos psicossociais incialmente encarados como estranhos e distantes da realidade, a ancoragem permite nomeá-los e alocá-los em categorias mentais conhecidas a partir das experiências cotidianas, estabelecendo-lhes valores sociais, de acordo com normas ou preceitos dos grupos humanos. Por sua vez, a objetivação atribui imagens ou símbolos, transferindo para o plano físico as elaborações do plano psíquico, na tentativa de materializá-las e, assim, aproximá-las da realidade. Esses processos ocorrem a partir de uma relação dialógica entre a natureza conceitual do objeto, que lhe atribui sentido, e a sua natureza figurativa, que lhe atribui um ícone^(8)^.

### Cenário do estudo e fonte de dados

Foi realizado no curso de graduação em enfermagem de uma universidade pública em Belém, capital do estado do Pará, Brasil, nos contextos geográfico e social da chamada Região Metropolitana de Belém, formada por outros sete municípios que se integram para fortalecer a organização, o planejamento e a execução de políticas públicas^(17)^.

Com duração de cinco anos e carga horária de 5.000 horas, o curso é operacionalizado em cinco séries e dez blocos semestrais, pois cada série se constitui por dois blocos, correspondentes ao 1º e 2º semestres. O corpo docente é formado por enfermeiros e profissionais de outras áreas, com títulos de especialista, mestre ou doutor e vinculação efetiva ou temporária, cujos componentes curriculares figuram em 11 departamentos. A coordenação de curso é assumida por enfermeiro docente, que realiza atividades na capital e no interior do estado^(18)^.

Participaram 37 estudantes matriculados nas cinco séries do curso, reatores à prova tuberculínica, considerando os que apresentaram enduração igual ou superior a 5 mm. A enduração resultou de testagem que ocorreu em estudo multicêntrico liderado pela instituição dos autores, em parceria com outras três universidades públicas, sediadas nas capitais dos estados do Amazonas, Piauí e Rio de Janeiro, visando determinar a prevalência de ILTB nesse público.

No município de Belém, a testagem ocorreu entre dezembro de 2022 e fevereiro de 2023, alcançando 269 participantes e 74 reatores (27,51%). Planejou-se incluir a metade dos reatores, entendendo que essa amostra seria numericamente representativa. Todavia, durante a pesquisa, os autores optaram por também considerar a saturação teórica, pois constitui importante elemento da abordagem qualitativa, sobretudo diante de um referencial teórico-metodológico específico. Assim, identificou-se a saturação quando os dados se mostraram satisfatórios para alcançar o objetivo e acréscimos provavelmente não alterariam a compreensão sobre o fenômeno representacional^(19)^. À medida que os depoimentos eram obtidos, verificava-se cuidadosamente se a saturação havia sido atingida.

Foram excluídos os reatores que não puderam ser localizados e os que manifestaram impedimentos para agendar entrevista, não havendo desistências.

### Produção dos dados

No intuito de obter autorização, o primeiro autor visitou a universidade para apresentar o projeto à coordenadora de curso, momento no qual pactuou a liberação de uma sala para realizar as entrevistas individuais, que ocorreram no período de março a julho de 2023. Os processos de formação desenvolvidos em grupo de pesquisa vinculado à instituição dos autores e as reuniões de orientação do curso de mestrado em enfermagem, no qual o autor estava matriculado, qualificaram-no para conduzir as entrevistas.

Com a lista de reatores, contendo nomes, dados telefônicos e informações sobre a prova tuberculínica, fornecida por pesquisadores do estudo multicêntrico, com a devida autorização dos estudantes, o autor os contactou por ligação telefônica ou aplicativo de mensagens, informando brevemente do que a pesquisa se tratava para convidá-los a participar. Os que aceitaram foram orientados a vir à sala reservada, em dia e horário agendados, na qual estavam apenas o autor e o participante, respeitando sua privacidade. Como estratégias para evitar a COVID-19 em ambiente fechado, disponibilizou-se álcool em gel 70%, informando-se a possibilidade de usar máscara.

Inicialmente, os objetivos, procedimentos, riscos e benefícios foram detalhados com o TCLE, esclarecendo-se dúvidas para obter o aceite formal. As entrevistas foram guiadas por roteiro semiestruturado, construído pelos dois primeiros autores e submetido à avaliação de quatro pesquisadoras da área de enfermagem, com sólida produção científica e experiência em orientar estudantes de graduação e pós-graduação *stricto sensu*. Entre elas, uma desenvolvia estudos de RS, e todas eram *experts* em temas relacionados às doenças transmissíveis, à educação superior e à saúde coletiva. Após a avaliação, duas referendaram o instrumento, e as demais teceram sugestões para fortalecer o seu conteúdo, as quais foram ponderadas, e os aspectos oportunos foram acatados. Por ter sido qualificado, optou-se por não submetê-lo a um pré-teste ou teste piloto.

No primeiro eixo do roteiro, investigou-se o perfil sociodemográfico e acadêmico, por meio de questões objetivas que abrangeram variáveis de pertença grupal, entendendo que os sujeitos falam a partir dos lugares que ocupam na sociedade, resultando na contextualização das RS^(11)^. Essas variáveis foram idade, sexo, período letivo, raça/cor de pele, religião, situação conjugal, número de filhos, percepção de bolsa de estudos, ocupação, renda familiar, município de residência, número de pessoas com quem residiam e, adicionalmente, o tamanho da enduração. No segundo eixo, investigou-se o fenômeno representacional com questões subjetivas que permitiram identificar experiências e reflexões acerca do tema. As entrevistas duraram de 20 a 50 minutos, e foram gravadas, no formato MP3, utilizando equipamento digital.

Como o autor integrou a equipe do estudo multicêntrico e operacionalizou a testagem, os estudantes o conheciam e estavam cientes que a pesquisa com reatores figurava como requisito para concluir o curso de mestrado. Ressalta-se que o conteúdo das entrevistas não foi partilhado com eles para o referendarem, pois isso comprometeria a espontaneidade dos depoimentos, valorizada como elemento fundamental para a TRS. Diante da completude dos resultados, as entrevistas não foram repetidas e não foram adotadas outras técnicas de produção.

### Organização e análise dos dados

Os dados do perfil sociodemográfico e acadêmico foram armazenados na versão 2013 do *Microsoft Office Excel* ^®^, criada pela *Microsoft Corporation*. Isso possibilitou a análise estatística descritiva, que identificou valores absolutos e relativos.

As respostas às questões subjetivas foram transcritas para gerar um *corpus*, seguindo as orientações técnicas e metodológicas da versão 0.7 (*alpha* 2) do *software Interface de R pour les Analyses Multidimensionnelles de Textes et de Questionnaires* (IRaMuTeQ^®^)^(20)^, criado pelo francês Pierre Ratinaud e com o qual realizou-se análise lexical em português, considerando que apresenta dicionários completos em vários idiomas^(21)^.

O *corpus* foi organizado em arquivo único, sem formatação (.*txt*), com codificação de caracteres no padrão *Unicode Transformation Format 8-bit*. Por utilizar funções estatísticas do *software* R e linguagem de programação *Python*, o IRaMuTeQ^®^ permite investigar dados textuais com rigor científico, a exemplo dos que são oriundos de um conjunto de transcrições sobre tema específico, como é o *corpus* deste estudo^(20)^. Além disso, por ser de acesso livre, o IRaMuTeQ^®^ tem sido largamente utilizado em pesquisas qualitativas^(21)^, inclusive com a TRS^(22)^.

Seu *menu* oferta cinco modalidades: estatísticas lexicográficas; especificidades e análise fatorial de correspondência; Classificação Hierárquica Descendente (CHD); análise de similitude; e nuvem de palavras. Entre elas, optou-se pela CHD, visando desmembrar o *corpus* em segmentos de texto (STs), que compuseram classes lexicais formadas por palavras dispostas verticalmente em um dendrograma, considerando representativas as que obtiveram p<0,0001. Os elementos semânticos dessas palavras foram apontados pelos STs nos quais elas se localizavam e que lhes deram sentido. Quatro valores estatísticos lhes foram atribuídos: frequência de STs que continham a palavra na classe (Fc); frequência total de STs que continham a palavra no *corpus* (Ft); percentual de STs que continham a palavra na classe (%), em relação à ocorrência no *corpus*; e força associativa da palavra, demonstrada pelo qui-quadrado (X^2^), que define sua posição ordinal na classe^(20)^.

Com a organização realizada pela CHD, muitas vezes não é possível utilizar o total de STs, motivo pelo qual autores reconhecem que, para uma análise confiável, o percentual mínimo de aproveitamento deve ser 75%^(21,23)^. Os dados foram interpretados e discutidos a partir de evidências da literatura científica sobre o tema e dos processos de ancoragem e objetivação, cujos conceitos foram apresentados no referencial teórico-metodológico.

## RESULTADOS

Entre os participantes, a idade variou de 19 a 40 anos, sendo que 28 (75,68%) estavam na faixa etária de 19 a 23 anos e 24 (64,86%) eram mulheres. No tocante aos períodos letivos, sete (18,92%) cursavam a 1ª série; cinco (13,51%), a 2ª; oito (21,62%), a 3ª; dez (27,03%), a 4ª; e sete (18,92%), a 5ª. Abrangendo as outras variáveis, 21 (56,76%) se declararam pardos; 17 (45,95%), evangélicos; e 32 (86,49%), solteiros. Além disso, 35 (94,59%) não tinham filhos; 22 (59,46%) não recebiam bolsa de estudos; 31 (83,78%) não exerciam atividades profissionais; e 23 (62,16%) declararam renda familiar entre dois e três salários mínimos mensais.

Todos residiam em municípios da Região Metropolitana de Belém, com uma a 18 pessoas vivendo no mesmo domicílio. Nesse contexto, um participante (2,70%) afirmou viver, em casa própria, com 13 pessoas, e outro (2,70%), com 18, entre agregados e familiares com diversos graus de parentesco. Todavia, grande parcela (n=13; 35,14%) afirmou residir com apenas três pessoas, entre as quais destacaram-se as do círculo familiar, principalmente mãe, pai e irmãos. Os tamanhos da enduração, gerada pela prova tuberculínica, variaram de cinco a 20 mm, com média de 11,70 mm, sendo que 13 (35,14%) apresentaram 10 mm.

Com 37 textos, o IRaMuTeQ^®^ gerou 1.939 STs pelo desmembramento da CHD, resultando no aproveitamento de 1.686 (86,95%), considerado satisfatório. Foram identificadas 67.619 ocorrências, que correspondem ao total de formas ou palavras, das quais 5.022 eram palavras distintas e 2.367 eram palavras com apenas uma frequência.

As partições do *corpus* originaram dois *subcorpora*, constituídos por sete classes lexicais, de modo que, no 1º *subcorpus*, alocou-se a classe 2, responsável por 281 STs (16,67%), e as outras seis foram alocadas no 2º *subcorpus*, nesta ordem: classe 5, responsável por 175 STs (10,38%); classe 6, responsável por 278 STs (16,49%); classe 7, responsável por 277 STs (16,43%); classe 1, responsável por 262 STs (15,54%); classe 3, responsável por 174 STs (10,32%); e classe 4, responsável por 239 STs (14,18%). Assim, as classes 2 e 3 foram, respectivamente, consideradas a maior e menor entre as sete, como mostra o dendrograma vertical da CHD, gerado pelo IRaMuTeQ^®^, com as palavras iniciais de cada classe (Figura 1).

**Figura 1:** Dendrograma vertical da Classificação Hierárquica Descendente, mostrando a lógica de partição do *corpus*, os percentuais de segmentos de texto (padronizados, pelo IRaMuTeQ^®^, com uma cada decimal após a vírgula) e as palavras iniciais das classes lexicais, Belém, Pará, Brasil, 2023

*DIP - doenças infecciosas e parasitárias; EPI - equipamento de proteção individual; ILTB - infecção latente da tuberculose.*

Essas palavras evidenciam um conjunto diversificado de pensamentos e práticas sociais relacionados às RS dos estudantes sobre o tema. Por esse motivo, as classes foram agrupadas em três eixos temáticos, constituindo os resultados da pesquisa original. Com base na complementariedade e convergência semântica entre as classes, os eixos foram assim nomeados e organizados: I) “Significando a ILTB e a TB: saberes, afetos e memórias”, com as classes 1, 3 e 4; II) “Ser reator: descobrindo, cuidando de si e do outro”, com as classes 2, 5 e 6; III) “Informar para melhor cuidar: desafios e possibilidades na educação superior”, com a classe 7.

Visando atender ao objetivo deste estudo, optou-se por destacar a classe 4, que constituiu o 2º *subcorpus* e apresentou 60 palavras representativas (p<0,0001), das quais 30 (50%) foram detalhadas na Tabela 1, por serem as mais representativas. Essa escolha se justifica tanto pelo grande volume de dados da pesquisa original, que inviabiliza sua adequada apresentação em apenas um manuscrito, quanto pela completude e relevância dos resultados da classe 4, que permitem explorá-la separadamente e com a devida profundidade teórico-metodológica.

**Tabela 1:** Detalhamento lexical da classe 4, Belém, Pará, Brasil, 2023

Nº	Palavras	Fc	Ft	%	X^2^
1	Tuberculose	133	510	26,08	85,15
2	Pneumonia	18	22	81,82	83,83
3	Ano	16	19	84,21	77,47
4	Tosse	23	38	60,53	68,65
5	Avô	22	37	59,46	63,76
6	Morar	17	27	62,96	53,68
7	Médico	20	36	55,56	51,77
8	Época	17	29	58,62	47,91
9	Tio	17	30	56,67	45,33
10	Diagnosticar	13	20	65,00	42,97
11	Nascer	7	7	100,00	42,56
12	Quadro	8	9	88,89	41,52
13	Suspeita	8	9	88,89	41,52
14	Casa	25	60	41,67	38,65
15	Acompanhar	10	14	71,43	38,04
16	Visitar	6	6	100,00	36,46
17	Lembrar	33	95	34,74	34,98
18	Morrer	14	26	53,85	34,16
19	Serviço_de_saúde	5	5	100,00	30,36
20	Sogro	6	7	85,71	29,57
21	Meu_Deus	9	14	64,29	29,14
22	Pegar	24	66	36,36	27,80
23	Pai	22	60	36,67	25,87
24	Lá	11	21	52,38	25,51
25	Óbito	4	4	100,00	24,28
26	Bairro	4	4	100,00	24,28
27	Recluso	4	4	100,00	24,28
28	Criança	8	13	61,54	24,16
29	Tratar	16	40	40,00	22,46
30	Família	25	78	32,05	21,48

*Fc - frequência de segmentos de texto que continham a palavra na classe; Ft - frequência total de segmentos de texto que continham a palavra no corpus; % - percentual de segmentos de texto que continham a palavra na classe, em relação à ocorrência no corpus; X^2^ - qui-quadrado.*

Essa classe apresentou STs que remetem às experiências não acadêmicas dos participantes no contato com o tema e às reflexões que empreenderam após o resultado reator, no intuito de melhor compreender em quais possíveis ocasiões se expuseram ao bacilo, ocasionando a ILTB.

Assim, foram desveladas diversas experiências com pessoas próximas, como amigos e familiares, ou com pessoas cujo relacionamento era distante e esporádico. Nesse contexto, destacaram-se as experiências familiares de adoecimento por TB ou outras doenças respiratórias, que ocorreram recentemente ou há vários anos, com avós, pais e tios, entre outros familiares.

Esse aspecto é amplamente ilustrado pelas palavras de maior força (“tuberculose” (X^2^=85,15) e “pneumonia” (X^2^=83,83)), associadas a palavras como “tosse” (X^2^=68,65), “avô” (X^2^=63,76), “médico” (X^2^=51,77), “época” (X^2^=47,91), “tio” (X^2^=45,33), “diagnosticar” (X^2^=42,97), “suspeita” (X^2^=41,52), “morrer” (X^2^=34,16), “sogro” (X^2^=29,57), “pai” (X^2^=25,87) e “recluso” (X^2^=24,28), como demonstram os excertos a seguir:

*Minha sogra esteve internada com suspeita de TB, e tinha tosse seca prolongada. Quando a gente procurou o serviço de saúde para internar, porque ela já tinha histórico de pneumonia, a gente perguntou para o médico se não era o caso de fazer uma pesquisa para ver se ela estava com TB. Ficou internada por conta disso e, graças a Deus, ele descartou a possibilidade de TB, mas permaneceu internada com pneumonia novamente.* (EIII-11)

*Meu tio adoeceu de TB em 2022, passou mal e teve que fazer uma cirurgia no pulmão. Na época, eu tinha contato com ele, porque vinha em minha casa. Tínhamos contato não no estágio mais grave da doença, porque depois* [que o quadro clínico agravou] *ele ficou recluso. Antes, nem ele sabia que estava doente, porque sempre foi assim, alguém que tinha doenças e não se preocupava. Não sei se meu pai pegou do meu tio, porque meu pai também tem TB, foi diagnosticado há pouco tempo e está fazendo o tratamento.* (EI-26)

*Na minha família, já conversamos que minha avó teve pneumonia, mas não se sabe se foi só pneumonia, porque, antigamente, não havia rastreamento. Ela trabalhava batendo açaí, não tinha um médico, e, quando fez uma tomografia, já era idosa e apresentava tosse crônica. Quando fez um raio X dos pulmões* [referindo-se à radiografia de tórax]*, já havia uma calcificação na base do pulmão, mas não morreu por essa causa. Isso foi apenas constatado, e a minha mãe associou ao suposto quadro de pneumonia que minha avó teve e que tratou com remédios caseiros.* (EIV-33)

Entre os que relataram ter conversado sobre a TB, ou desenvolvido outra forma de contato com o tema por meio de mídias audiovisuais (como a televisão), antes de ingressarem no curso ou fora do ambiente acadêmico, percebeu-se a associação da doença com eventos ou movimentos históricos, como o Romantismo, movimento artístico-cultural que data dos séculos XVIII e XIX, e a Segunda Guerra Mundial, ocorrida no século XX.

Por influência das experiências escolares no ensino médio, alguns participantes ancoraram a ocorrência da TB a condições de privação e sofrimento, pois fragilizam o organismo, predispondo-o ao adoecimento. Essa ideia foi objetivada pelo uso das expressões “mal do século” e “Alemanha Nazista”, que remetem a momentos de crise historicamente documentados e perpetuados, configurando a projeção imagética de tais condições:

*Outra memória que tenho é da disciplina de literatura na escola, em que tinha o Romantismo, 2ª geração, e tinha o mal do século, porque era muito comum os escritores morrerem de TB. O sofrimento influenciava a escrita deles, como tratavam a história, como viam o mundo dentro da narrativa. Isso me gerou curiosidade sobre a doença.* (EI-10)

*Alguns filmes me remeteram ao Romantismo, em que as pessoas morriam pela TB, que era o mal do século. Pessoas pegavam* [a doença]*, iam para as montanhas buscar ar puro, tentando melhorar, mas muitas evoluíam a óbito. Lembro que via isso em filmes e também estudando literatura sobre o Período Romântico no ensino médio.* (EII-12)

*Houve muitos casos de TB na época da Alemanha Nazista, nos campos de concentração, justo porque as pessoas tinham resistência* [imunidade] *baixa, não se alimentavam, não viviam em condições decentes e, por isso, tinham mais possibilidade de adoecer.* (EI-26)

Todos reconheceram que, em algum momento, foram expostos ao bacilo, dentro ou fora do ambiente acadêmico, embora não pudessem afirmar em que momento a infecção ocorreu. Contudo, na tentativa de encontrar um nexo para o resultado reator, os participantes rememoraram situações que, na concepção deles, podem ter causado ou propiciado a ILTB, ancorando-a em três fatores principais, que ocorreram de forma conjunta ou isolada: 1) contato com pessoas que estavam com TB; 2) contato com pessoas que apresentavam outras doenças respiratórias ou estavam sob suspeita de TB; 3) exposição a ambientes com alto risco biológico, como unidades de saúde e hospitais, para realizar atividades durante o curso, mas também, no caso de alguns participantes, para receber atendimento médico, principalmente durante a infância ou adolescência.

Essa atitude reflexiva é denotada pela palavra “lembrar” (X^2^=34,98), associada a palavras como “ano” (X^2^=77,47), “morar” (X^2^=53,68), “casa” (X^2^=38,65), “serviço de saúde” (X^2^=30,36), “criança” (X^2^=24,16) e “família” (X^2^=21,48), remetendo aos locais e às circunstâncias em que a exposição e o contágio possivelmente ocorreram:

*Minha bisavó, que faleceu há muitos anos, teve TB e cuidei dela durante três anos, nos últimos anos de vida. Cuidei após o tratamento! Antes da doença, ela já morava em minha casa, então acredito que tive contato com a bactéria por causa dela.* (EII-4)

*Nas aulas práticas, a gente teve contato, algumas vezes, com pessoas que tinham suspeitas de estarem doentes, mas nunca tivemos a confirmação. Por isso, não sei se adquiri ILTB estagiando em serviços de saúde ou se adquiri em outro lugar, como no transporte público. Quando eu era mais jovem, tinha muita alergia, então ia muito ao médico. Além disso, ia ao ortopedista, pois sempre tive problemas de coluna.* (EIII-7)

*Quando era criança, eu vivia no hospital* [buscando atendimento médico]*. Só parei de entrar em hospital quando tinha uns 12 anos. Desde que nasci, tive muitos princípios de pneumonia, e vivia internada. Como sempre tive problemas respiratórios, talvez, por conta disso, de estar no hospital, posso ter sido sensibilizada* [infectada]*, ou até em uma rua qualquer, posso ter entrado em contato com a bactéria.* (EI-13)

*Quando soube que havia suspeita de TB na minha avó, pensei assim: “Meu Deus, creio que mais pessoas da família podem estar contaminadas, inclusive eu, por ter tido contato com meu pai”* [que contraiu a doença]*. Por não ter apresentado nenhum sintoma, não fui logo buscar uma investigação para mim.* (EIV-29)

## DISCUSSÃO

Baseando-se na memória social, os resultados apresentam as experiências não acadêmicas em torno do tema e a atitude reflexiva dos participantes para vincular o resultado reator a eventos pregressos. Entende-se que a rememoração das experiências e tal atitude reflexiva contribuem para naturalizar esse evento inusitado, isto é, ser reator à prova tuberculínica e viver com ILTB.

No campo dos fenômenos representacionais, a naturalização ocorre para estabelecer identidade sociocultural dos sujeitos com o objeto representado, remetendo aos processos de ancoragem e objetivação^(24)^, que não se desdobram, necessariamente, nessa ordem, pois são dinâmicos e interdependentes, ocorrendo de forma simultânea^(25)^.

Uma vez que o objeto é ancorado, isto é, integrado a um sistema de conhecimentos preexistentes, a objetivação faz com que seja percebido como real pelo indivíduo e por sua coletividade^(9)^. Assim, para cumprir seu propósito, a ancoragem se desdobra em três momentos: atribuição de sentido, por meio de um conjunto de significados preexistentes; instrumentalização do saber, que corresponde à funcionalidade; e transformação icônica, momento em que as RS se tornam familiares. Por sua vez, a objetivação expressa três aspectos: seleção e descontextualização, em que certos elementos são destacados da rede de informações; construção do núcleo figurativo ou imaginante; e naturalização, identificando o objeto na realidade^(26)^.

Evidenciou-se o adoecimento de familiares por TB ou outras condições respiratórias, aflorando, simultaneamente, afetos e memórias em torno da doença como fortes marcas das RS, haja vista o acesso cognitivo a informações que aludiam ao adoecimento de entes queridos.

Dialogando com esse resultado, estudos analisaram RS de mulheres quilombolas da Amazônia paraense sobre o cuidado em saúde, apresentando relatos de adoecimento entre familiares e outros membros da comunidade. Apontaram o modo como as mulheres cuidavam dessas pessoas e as explicações que justificavam as ações empreendidas^(27,28)^. Construiu-se a figura-tipo da “supermãe”, representando a ideia-conceito e a projeção imagética da mulher com diferentes atributos e qualidades, que cuida do ambiente doméstico, da família e da comunidade, com afeto e dedicação^(27)^.

Influenciados pelos conhecimentos adquiridos no ensino médio e pelas vivências que construíram nesse contexto, os participantes verbalizaram a expressão metafórica “mal do século” e o termo “Alemanha Nazista” para se reportar à TB, às suas causas e aos seus efeitos multilaterais, objetivando-a como entidade histórica e mazela social. Nesse entendimento, a doença é produto de condições de vida sub-humanas, especialmente ligadas à pobreza ou baixa renda, e a situações de apoio social inadequadas, como reiterado por vários estudos nacionais^(29,30)^ e internacionais^(31,32)^.

Todavia, é oportuno ressaltar que o Brasil dispõe de vários programas sociais governamentais de suporte e de transferência de renda, mesmo com entraves operacionais relacionados, entre outros fatores, ao baixo conhecimento e às limitações impostas pela governabilidade administrativa dos profissionais de saúde e da assistência social, como por vezes ocorre entre os que atuam na APS^(29,33)^. Alguns desses programas são o Benefício de Prestação Continuada e o Programa Bolsa Família, além de outras iniciativas, que incluem, por exemplo, aposentadoria por invalidez e para pessoas de baixa renda, isenção de impostos sobre rendimentos relativos a aposentadorias ou pensões, auxílio-doença, e auxílio financeiro para alimentação e transporte^(34)^.

Embora não sejam específicos a pessoas com TB, esses programas são direcionados aos grupos humanos que enfrentam condições de vida ou de saúde desafiadoras, o que necessariamente inclui o adoecimento por TB. Isso tem como finalidade mitigar as repercussões socioeconômicas sobre o orçamento do indivíduo e de sua família, incentivar a continuidade do tratamento para obter a cura, e fortalecer a qualidade de vida^(30)^.

Reafirmando a necessidade de promover adequado suporte social no contexto da TB, o Ministério da Saúde elaborou um guia para esclarecer diversos aspectos, a exemplo dos mecanismos de proteção que existem no território nacional e como ampliá-los, além das possibilidades para integrar as ações do Sistema Único de Saúde (SUS) e do Sistema Único de Assistência Social (SUAS), e da importância de a sociedade civil fortalecer essa integração^(34)^. Por isso, de forma conjunta, o Ministério da Cidadania e o Ministério da Saúde publicaram a Instrução Operacional nº 1, de 26 de setembro de 2019, reforçando as articulações entre SUS e SUAS para viabilizar o enfrentamento coletivo da TB^(35)^.

Posto que o homem constrói seus pensamentos com base na teia de conhecimentos que tem acesso^(8)^, as formas tradicionais de pensamento contribuem para ancorar um novo conhecimento (neste caso, a ILTB entre os que com ela vivem) em categorias socialmente elaboradas e partilhadas, permitindo aos sujeitos compreendê-lo^(13,36)^, razão pela qual entende-se que a TB constituiu pano de fundo no qual os estudantes construíram e expressaram suas RS. Em vista disso, a ancoragem situa o papel da memória social na construção simbólica da realidade para que o homem a entenda e nela atue. Assim, a ciência interage com o senso comum, sem que essa interação necessariamente provoque contraposições em relação às formas tradicionais de pensamento^(36)^.

Ainda sobre a construção psicossocial da TB como entidade histórica, sabe-se que o século XIX representou o momento mais crítico da doença no mundo e no Brasil, país em que o número de mortos provocado por ela era incontável, justificando sua denominação como “mal do século”. Visto que, naquele momento, as medidas de proteção individual e coletiva eram incipientes e muito limitadas, a TB se tornou um problema de saúde pública ainda maior entre os que tinham hábitos considerados desregrados, pois eram mais vulneráveis ao adoecimento pelo modo de vida boêmio, permeado por excessos e vícios, além do desejo de “morrer de amor” diante dos sentimentos amorosos não correspondidos^(37)^.

Como evidenciado por alguns participantes, esse era o caso dos poetas byronianos, pois quase todos os da segunda fase do Romantismo no Brasil morreram jovens, em decorrência da TB. Exemplos disso são Álvares de Azevedo (1831-1852), que morreu aos 21 anos, Casimiro de Abreu (1839-1860), que também morreu aos 21, e Castro Alves (1847-1871), que morreu aos 24 anos^(37)^. A ancoragem da TB à Segunda Guerra Mundial também encontra amparo na literatura, pois a doença era compreendida como risco de guerra pelos soldados, do qual era necessário se defenderem e defenderem à sua pátria^(38)^.

Reconhecendo que tiveram contato prévio com o bacilo e que, por isso, vivem com ILTB, os estudantes refletiram sobre a possibilidade de terem sido expostos em situações de contato com pessoas acometidas por TB ou por outras doenças respiratórias, ou mesmo em ambientes de risco, sobretudo em serviços de saúde, nos quais realizaram atividades durante o curso ou buscaram algum tipo de atendimento durante a infância ou adolescência. Essa noção epidemiológica de risco e exposição é referendada pelo Ministério da Saúde^(1,3)^ e por diferentes estudos^(2,6,7)^.

Apesar de os conhecimentos adequados sobre o tema serem fundamentais no cotidiano pessoal e acadêmico desse público, vislumbrando a atuação profissional futura, pesquisa realizada com 60 estudantes de enfermagem de uma universidade federal localizada no município de Três Lagoas, Mato Grosso do Sul, identificou conhecimentos limitados entre estudantes com menor tempo de estudo universitário e que não tinham contato prévio com o tema, contrastando com os conhecimentos melhor estruturados entre aqueles que obtiveram informações em serviços de saúde. Isso apontou que estratégias de ensino-aprendizagem precisam ser fortalecidas, repensadas ou reestruturadas na formação em enfermagem, oportunizando o compartilhamento oportuno e a apreensão de informações sobre um tema tão relevante^(39)^.

Essa ponderação considera o atual cenário epidemiológico da TB no Brasil^(40)^ e no mundo^(4)^, reforçado pela alta prevalência de ILTB e por diversos fatores estruturais que, juntos, mantêm a doença como grave problema de saúde pública^(4,40-43)^. Também não se deve esquecer que o tema ainda é envolto por medos, estigmas e preconceitos, cristalizados ao longo do tempo e manifestos no cotidiano dos grupos humanos, inclusive durante a atuação profissional de enfermeiros na APS^(10)^.

Corroborando essa reflexão, pesquisa no município da Bolgatanga, região superior leste de Gana, foi realizada com três grupos focais, totalizando 19 participantes, dos quais 13 viviam com TB e seis não viviam. Evidenciou-se forte manifestação de estigmas da comunidade contra os que viviam com TB, pois pessoas sem a doença apresentavam receio de se aproximarem deles, reforçando condições de isolamento e distanciamento social^(44)^.

Isso obstaculiza o controle efetivo por meio das ações que devem ser realizadas com o indivíduo, sua família e sua comunidade, o que abrange investigar, tratar e acompanhar casos de ILTB entre os contatos^(1,3)^, mas também aponta que estratégias de educação em saúde precisam ser adotadas racionalmente. Faz-se tal ponderação visando transformar saberes e práticas no contexto comunitário para além das estratégias de ensino-aprendizagem que possibilitam robustecer tanto a formação acadêmica na educação superior quanto a formação profissional no cotidiano dos serviços de saúde.

### Limitações do estudo

Visto que o estudo foi realizado no curso de graduação em enfermagem de uma instituição de ensino superior pública no município de Belém, configurando realidade específica, entende-se que os dados foram determinados por aspectos que contextualizam as RS, como fatores educacionais, geográficos e socioculturais. Isso culminou na impossibilidade de considerar que os resultados, na íntegra, têm potencial para convergir com as realidades pessoal e acadêmica enfrentadas por estudantes de enfermagem que vivenciam o curso em outras instituições de ensino superior públicas ou privadas no estado do Pará, em outros estados da região Norte ou no restante do território brasileiro.

Todavia, ao menos parcialmente, os resultados podem convergir com as realidades desses sujeitos, pois o fato de serem estudantes os aproxima, auxiliando-os a melhor compreender o tema e compartilhá-lo por meio das experiências em sala de aula e fora dela.

### Contribuições para as áreas de enfermagem, saúde ou políticas públicas

Baseando-se nos resultados e nas suas discussões, autoridades públicas no campo da saúde e gestores acadêmicos podem refletir sobre a necessidade de propor, manter ou reestruturar medidas que ampliem as condições de biossegurança dos ambientes nos quais os estudantes de enfermagem realizam atividades internas e externas à universidade. Nesse contexto, destacam-se os serviços de saúde, como os que prestam assistência a pessoas com TB e seus contatos, no intuito de prevenir a ocorrência de ILTB entre estudantes.

Diante das reflexões aqui apresentadas, espaços de discussão podem ser fomentados na comunidade acadêmica e entre profissionais de saúde, visando esclarecer aspectos relacionados ao tema e democratizar o seu compartilhamento oportuno. Isso corrobora as RS como formas de conhecimento que possibilitam melhor compreender a realidade na qual se está imerso, a fim de agir dentro dela, em um constante movimento de retroalimentação cognitiva e perceptiva, o que reafirma o caráter dinâmico e pragmático de tais representações.

## CONSIDERAÇÕES FINAIS

Este estudo desvelou experiências e reflexões de estudantes de enfermagem sobre a ILTB, expressadas por meio das RS. Nesse contexto, foram relatadas diversas experiências não acadêmicas, sobretudo de adoecimento por TB ou outras condições respiratórias envolvendo pessoas de suas famílias, além de situações do cotidiano escolar no ensino médio, por meio das quais tiveram contato com o tema. Destacaram-se as reflexões empreendidas após o resultado reator, que objetivavam melhor compreender as circunstâncias nas quais possivelmente se expuseram ao bacilo e foram contaminados, estabelecendo nexo para justificar essa condição.

Visando proteger os estudantes, tanto em seu cotidiano pessoal quanto no acadêmico, as ações de biossegurança requerem parcerias colaborativas entre os setores de saúde e educação, integrando seus representantes para que esse fim seja alcançado. Contudo, os resultados demonstram que também é necessário fortalecer as ações de cuidado, especialmente na APS, para que os doentes sejam identificados, tratados e acompanhados de maneira oportuna, rompendo a cadeia de transmissão para evitar maior contingente de infectados e de novos adoecidos.

Por evidenciarem que a integração entre esses setores é necessária, espera-se que os resultados mobilizem estudantes e docentes-pesquisadores da área de enfermagem e de áreas correlatas, como educação, psicologia e saúde coletiva, a investirem no tema, para que outros estudos sejam realizados, abrangendo cenários e objetos inerentes à assistência, à gestão dos serviços de saúde, ao ensino, à pesquisa e à participação política de lideranças sociais em instâncias de poder e governança. A TRS pode fundamentar a concepção, o desenvolvimento e a interpretação desses estudos, constituindo oportunidade para romper possíveis fragilidades teórico-metodológicas.

Assim, será possível compreender aspectos ainda não investigados ou pouco elucidados, bem como ampliar a disseminação do tema a partir de publicações em periódicos científicos e em outros veículos confiáveis e da utilização culturalmente adequada desses materiais por profissionais qualificados. Isso tem como intuito transformar, em algum grau, a realidade dos grupos humanos que são por eles assistidos, sobretudo diante dos desafios impostos pela ILTB.

## Data Availability

Os dados de pesquisa só estão disponíveis mediante solicitação.
